# Enhanced Absorption Study of Ginsenoside Compound K (20-*O*-*β*-(D-Glucopyranosyl)-20(S)-protopanaxadiol) after Oral Administration of Fermented Red Ginseng Extract (HYFRG*™*) in Healthy Korean Volunteers and Rats

**DOI:** 10.1155/2016/3908142

**Published:** 2016-07-19

**Authors:** Il-Dong Choi, Ju-Hee Ryu, Dong-Eun Lee, Myoung-Hee Lee, Jae-Joong Shim, Young-Tae Ahn, Jae-Hun Sim, Chul-Sung Huh, Wang-Seob Shim, Sung-Vin Yim, Eun-Kyoung Chung, Kyung-Tae Lee

**Affiliations:** ^1^R&BD Center, Korea Yakult Co., Ltd., 418-12 Gomae-dong, Giheung-gu, Yongin-si, Gyeonggi-do 449-901, Republic of Korea; ^2^Department of Pharmaceutical Biochemistry, College of Pharmacy, Kyung Hee University, 1 Hoegi-dong, Dongdaemun-gu, Seoul 130-701, Republic of Korea; ^3^Department of Life and Nanopharmaceutical Science, College of Pharmacy, Kyung Hee University, 1 Hoegi-dong, Dongdaemun-gu, Seoul 130-701, Republic of Korea; ^4^Graduate School of International Agricultural Technology, Institute of Green Bio Science & Technology, Seoul National University, Pyeongchang 232-916, Republic of Korea; ^5^Kyung Hee Drug Analysis Center, 1 Hoegi-dong, Dongdaemun-gu, Seoul 130-701, Republic of Korea; ^6^Medical Center, Kyung Hee University, 1 Hoegi-dong, Dongdaemun-gu, Seoul 130-701, Republic of Korea; ^7^Department of Pharmacy, College of Pharmacy, Kyung Hee University, Seoul 02447, Republic of Korea

## Abstract

To evaluate the pharmacokinetics of compound K after oral administration of HYFRG and RG in humans, an open-label, randomized, single-dose, fasting, and one-period pharmacokinetic study was conducted. After oral administration of a single 3 g dose of HYFRG and RG to 24 healthy Korean males, the mean (±SD) of AUC_0–*t*_ and *C*_max_ of compound K from HYFRG were 1466.83 ± 295.89 ng·h/mL and 254.45 ± 51.20 ng/mL, being 115.2- and 80-fold higher than those for RG (12.73 ± 7.83 ng·h/mL and 3.18 ± 1.70 ng/mL), respectively; in case of Sprague Dawley rats the mean (±SD) of AUC_0–*t*_ and *C*_max_ of compound K from HYFRG was 58.03 ± 32.53 ng·h/mL and 15.19 ± 10.69 ng/mL, being 6.3- and 6.0-fold higher than those from RG (9.21 ± 7.52 ng·h/mL and 2.55 ± 0.99 ng/mL), respectively. *T*_max_ of compound K in humans and rats was 2.54 ± 0.92 and 3.33 ± 0.50 h for HYFRG and 9.11 ± 1.45 and 6.75 ± 3.97 hours for RG, respectively. In conclusion, the administration of HYFRG resulted in a higher and faster absorption of compound K in both humans and rats compared to RG.

## 1. Introduction

Ginseng (the root of* Panax ginseng* C. A. Meyer), a member of the Araliaceae family, is traditionally considered one of the most important medicinal plants. Over the last two decades, ginseng has been widely used as a dietary supplement and for therapy not only in Asian countries but also in the United States and Europe. Ginseng has a wide range of pharmacologic activities such as anti-inflammatory, antidiabetic, and anticancer activities [1–4]. These effects are attributed to its pharmacologically active compounds known as ginsenosides. Ginsenoside compound K (IH-901, 20-*O*-*β*-(D-glucopyranosyl)-20(S)-protopanaxadiol) is an active metabolite found in human plasma after oral administration of protopanaxadiol ginsenosides Rb1, Rb2, Rc, and Rd. Protopanaxadiol ginsenosides are metabolized to ginsenoside compound K by the intestinal microflora in humans [5–11]. Compound K has been reported to exhibit various pharmacologic activities* in vitro* and* in vivo* as well as anticancer effects [5–13]. Based on these pharmacologic activities, one way to promote the medical use of compound K is to increase its bioavailability.

The components of ginsenosides are affected by the processing method of* Panax ginseng* roots as in case of white and red ginseng. Red ginseng is produced by steaming raw white ginseng, resulting in the formation of active compounds such as Rg3, Rh4, and Rf2 in red ginseng, which have more potent pharmacologic activity than those in white ginseng [14, 15]. Previous studies have reported several methods for the transformation of the major ginsenosides to the minor ginsenosides, including acid hydrolysis [16], intestinal bacterial flora [17], and alkaline cleavage [18]. However, most of these physical and chemical methods are accompanied by inevitable and undesirable side reactions [19]. These problems associated with physical and chemical reactions could be avoided by using optimized microbial or enzymatic conversion methods because these methods use milder reaction conditions and are more environmentally compatible.

According to previous studies, although not present in the original ginseng preparation, compound K was detected in plasma and urine after oral administration of ginseng preparation in humans [16, 20, 21]. Previously, we reported the pharmacokinetic parameter estimates of IH-901 from fermented* Panax ginseng* are different from those of nonfermented ginseng where IH-901 is only formed through fermentation by the intestinal microflora [22]. However, no published data are available regarding the comparative pharmacokinetics of compound K from fermented Korean red ginseng (HYFRG) and nonfermented red ginseng (GR) extract in humans or in experimental rats. Based on that background, this study aimed to compare the pharmacokinetics of compound K after oral administration of HYFRG and RG extracts to healthy Korean subjects (*n* = 24) and experimental rats (*n* = 20) using an LC-MS/MS method for determination of compound K concentrations in human and rat plasma. For that purpose red ginseng was fermented in order to increase the amount of compound K, hence containing 3.65 mg of compound K/g, whereas nonfermented red ginseng was devoid of compound K.

## 2. Materials and Methods

### 2.1. Materials and Reagents

Compound K (purity > 98%) was obtained from Ambo Institute (Daejeon, Republic of Korea), and ginsenoside Rb1 (purity > 93%) and ginsenoside Rg1 (purity > 93%) were purchased from Chromadex (CA, USA). R-amlodipine (purity > 95%) used as an internal standard (IS) was obtained from HL Genomics (Yong-in, Kyunggi-do, Republic of Korea). Cytolase PCL5 and Rapidase C80Max were purchased from DSM Food Specialties (MA Delft, Netherlands), and Sumizyme AC was obtained from Shin Nihon Chemical (Aichi, Japan). HPLC grade of methanol, MTBE, and methylene chloride were purchased from J. T. Baker (Philipsburg, NJ, USA). A Milli-Q® (Bedford, MA, USA) water purification system was used to produce purified water for HPLC. All other chemicals and solvents were of the highest analytical grades available. Sodium acetate and acetic acid were purchased from Sigma (St. Louis, MO, USA).

### 2.2. Preparation of Red Ginseng and Fermented Red Ginseng Extracts

Six-year-old fresh ginseng (*Panax ginseng* C. A. Meyer) rootlet was purchased from Ginseng Nonghyup (Punggi, Republic of Korea). Red ginseng (RG) was made by steaming fresh ginseng at 95–100°C for 2 h and drying at 55–60°C. A voucher specimen (KGC040825) was deposited at the herbarium of Korea Yakult Research Institute (Yongin, Republic of Korea). Dried red ginseng (1 kg) was extracted in 20 L of 50% ethanol in water and concentrated with a vacuum concentrator. The red ginseng extract was incubated with three edible enzyme solutions containing 0.8% Cytolase PCL5, 0.8% Sumizyme AC, and 0.8% Rapidase C80Max at 50°C for 72 h and then fermented by* Lactobacillus sakei* HY7802. The reactants were heated at 100°C for 5 min to inactivate the enzyme; subsequently, the fermented RG diluent was evaporated to concentrate and designated as HYFRG. These samples were stored at 4°C and used when necessary. The amounts of ginsenosides Rb1, Rg1, and Compound K were 6.88, 1.33, and 0 mg/g in RG and 0, 4.12, and 3.65 mg/g in HYFRG, respectively.* Lactobacillus sakei* HY7802 (HY7802) was isolated and selected from Kimchi which is fermented cabbage widely consumed in Republic of Korea. HY7802 was grown in deMan-Rogosa-Sharpe (MRS) medium at 37°C for 18 h. The identity of HY7802 was confirmed by DNA sequencing of the 16S rRNA.

### 2.3. HPLC Analysis of Ginsenosides

The RG and HYFRG samples were extracted with 10-fold volume of methanol, filtered through a 0.45 *μ*m membrane, and then analyzed by an Agilent 1200 series HPLC system (Agillent, Foster City, CA, USA) using Discovery C_18_ column (250 × 4.6 mm, 5 *μ*m, Sigma-Aldrich, MO, USA) under UV detector at 203 nm. The injection volume was 5 *μ*L. The mobile phase consisted of acetonitrile (solvent A) and water (solvent B) applied by gradient at a flow rate of 1.6 mL/min. Gradient conditions were as follows: solvent A/solvent B (15/85, 20/80, 39/61, 48/52, 70/30, 90/10, 90/10, 15/85, and 15/85) with run times (0–5, 5–17, 17–57, 57–70, 70–80, 80–82, 82–92, 92–94, and 94–100 min), respectively.

### 2.4. LC-MS/MS Instrumentation and Chromatographic Conditions

**Figure 1:**
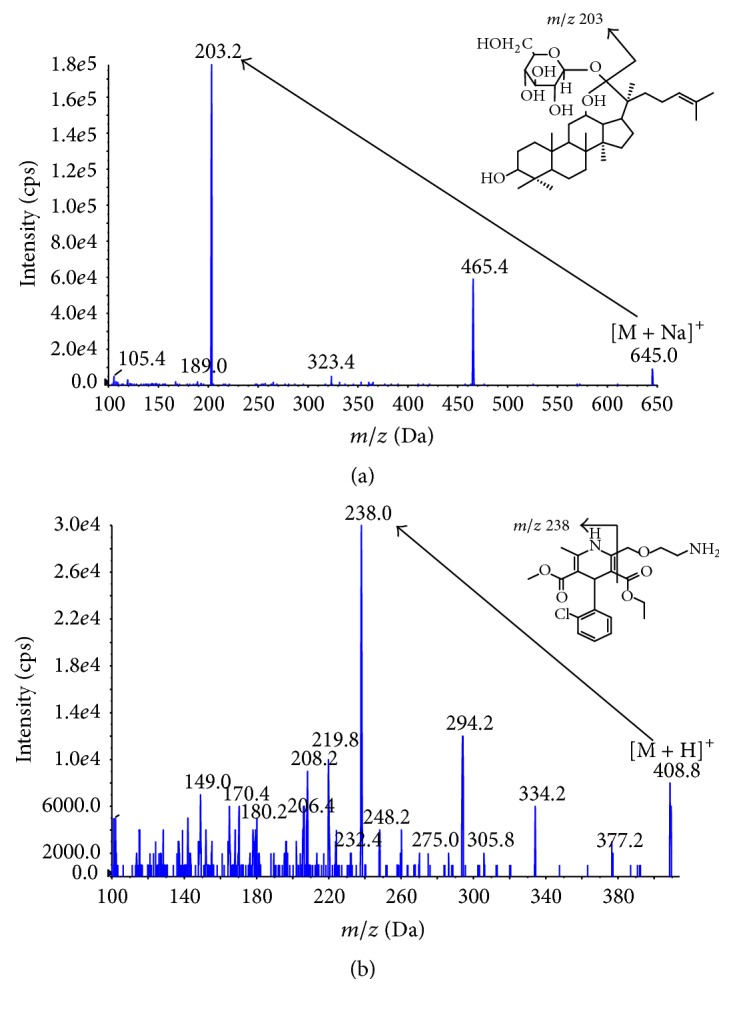
Full scan product ion spectra of (a) compound K and (b) R-amlodipine (IS).

Chromatographic analysis was performed using an Agilent 1200 series HPLC system (Agilent, Foster City, CA, USA). Compound K and IS were separated on a Phenomenex Luna C_18_ column (50 × 2.0 mm ID, 3 *μ*m; CA, USA) maintained at 40°C. The isocratic mobile phase consisted of methanol-10 mM sodium acetate buffer adjusted to pH 4 with acetic acid (93 : 7, v/v) and was filtered through a 0.22 *μ*m filter and degassed before use. The mobile phase was delivered at a flow rate of 0.2 mL/min and injection volume was 3 *μ*L. An API 3000 mass spectrometer (AB Sciex, Foster City, CA, USA) with a TurboionSpray interface operating in positive ionization mode was used for multiple reaction monitoring (MRM) mode. The mass spectrometer was operated at unit resolution for both Q1 and Q3 in MRM mode, with a dwell time of 200 msec per MRM channel. The MS/MS transitions (*m*/*z*) in positive ion mode were 645.0 → 203.2 for compound K and 408.89.1 → 238.0 for R-amlodipine (Figure 1), respectively. Nitrogen was used as a nebulizer and collision gas. TurboionSpray voltage was set to 5500 V, the curtain gas to 15 psi, and the collision activated dissociation gas to 30 psi. DP, EPl, and CXP were set at 120 eV, 12 eV, and 4 eV for compound K and 61 eV, 4.0 eV, and 6.0 eV for R-amlodipine, respectively. Data were processed using Analyst 1.5 (Foster City, CA, USA).

#### 2.4.1. Sample Preparation

Stock solutions of compound K and IS were prepared separately by dissolving the accurately weighed compounds in methanol for a final concentration of 1000 *μ*g/mL. The prepared stock solutions were stored in −80°C when not in use. Working solutions of compound K were prepared by serial dilution of the stock solution in methanol. Calibration standards were prepared by spiking 10 *μ*L of the appropriate standard solutions to 190 *μ*L of blank plasma for concentrations of 1, 5, 10, 50, 100, 500, and 1000 ng/mL. QC samples were prepared in the same manner as the calibration standards, for low, middle, and high concentrations of 3, 300, and 800 ng/mL. IS working solution (5 *μ*g/mL) for routine use was freshly prepared by diluting IS stock solution in methanol. The standard and QC samples were extracted on each analysis run using the same procedure described below.

All frozen subject samples, calibration standards, and QC samples were thawed at room temperature prior to analysis. An aliquot of 200 *μ*L plasma sample was transferred to a polypropylene tube followed by adding 20 *μ*L of IS solution (5 *μ*g/mL) and vortex mixing for 1 min. A single step liquid-liquid extraction (LLE) was adopted to extract compound K and IS from the human plasma. For LLE, 1.5 mL of methyl-*tert*-butyl-ether (MTBE): methylene chloride (8 : 2, v/v) was added to each tube followed by vortex mixing for 10 min. The well mixed solutions were centrifuged at 20,000 ×g for 10 min at 4°C, and 1.3 mL of the upper organic layer was transferred to a new tube and evaporated under a gentle steam of nitrogen at 40°C. The residues were reconstituted with 100 *μ*L of methanol followed by centrifugation at 20,000 ×g for 10 min at 4°C before analysis. An aliquot of 3 *μ*L was used for injection in the LC-MS/MS system.

#### 2.4.2. Method Validation

The LC-MS/MS method to determine compound K in human and rat plasma was validated and established in terms of specificity, sensitivity, linearity, accuracy, precision, recovery, matrix effect, and stability according to the bioanalytical method validation guidelines published by Korea Food and Drug Administration (KFDA) and USFDA [23, 24]. The specificity of the method towards endogenous plasma matrix substances was assessed in double blank human plasma (without compound K and IS). Double blank samples from six different lots of human blank plasma were tested for the presence of endogenous substances, which may interfere with compound K and IS using described above procedures and chromatographic/spectroscopic conditions. Then, the chromatograms obtained from the sample spiked with compound K at lower limit of quantitation (LLOQ) and IS and actual plasma samples from a volunteer after oral administration of compound K were compared with those obtained from blank samples. The calibration curve was constructed by plotting the peak area ratios (*y*) of compound K to IS against the spiked concentrations (*x*) of compound K. Linearity was assessed by weighted (1/*x*) least squares regression analysis. LLOQ was defined as the lowest concentration on the calibration curve. It should be quantified with an acceptable bias within ±20% and a CV no greater than 20%. The LLOQ was determined as the concentrations with a signal to noise (S/N) ratio of 10. The intra- and interday precision and accuracy assessments were performed on the same day (*n* = 5, at each concentration) and on three consecutive days at four concentrations (1, 3, 300, and 1000 ng/mL). Precision is expressed as a coefficient of variance (CV) at each concentration. The accuracy of the assay was defined as a percentage of the measured concentration over the theoretical concentration. The acceptance criterion recommended by KFDA and USFDA for each back-calculated standard concentration is 20% deviation from the nominal for LLOQ and 15% for all others (KFDA 2013, USFDA 2013). The relative recovery, absolute matrix effect, and process efficiency were determined at three QC concentrations (3, 300, and 800 ng/mL, *n* = 3) of compound K and IS (500 ng/mL, *n* = 9). The relative recovery was measured by comparing the peak areas obtained from plasma samples spiked before extraction with those from plasma samples spiked after extraction. The absolute matrix effect was measured by comparing the peak response of plasma samples spiked after extraction with that of pure standards containing equivalent amounts of compound K and IS prepared in mobile phase. The process efficiency wasmeasured by comparing the peak response of plasma samples spiked before extraction with that of the pure standards containing equivalent amounts of compound K and IS prepared in mobile phase.

#### 2.4.3. Cross Validation of Human and Rat Blood

According to the KFDA Guidelines on Bioanalytical Method Validation [23], different matrices may result in different outcomes between the study sites. Due to the scarcity of rat blood, the method development and validation of the LC-MS/MS assay were performed using human whole blood. A cross validation was performed by analyzing the rat blood spiked with analytes at LLOQ, low, medium, and high concentration levels (3, 300, and 800 ng/mL) using calibration standards and quality controls prepared in human whole blood in order to check whether the same results (±15% variation) can be obtained in both matrices.

### 2.5. Pharmacokinetic Study in Healthy Volunteers

This was an open-label, randomized-sequence, single dose, fasting, single-period pharmacokinetic study. Healthy Korean male volunteers aged 20–45 years were enrolled in the study. Study participants underwent screening examinations, and written informed consent was obtained from all subjects. This study was approved by the institutional review board of Kyung Hee University Hospital (KMC IRB1411-01) prior to the initiation of any study procedure. All study procedures were conducted in compliance with the principles of Declaration of Helsinki and Korean Good Clinical Practice guidelines. Subjects were randomly assigned to receive fermented red ginseng or nonfermented red ginseng in the first period. Subjects were admitted to Kyung Hee University Clinical Research Center at 5 PM on the day before the administration of red ginseng. All participants had a standardized dinner, and no food intake was permitted after 8 PM. The next day, subjects received 3 g of either fermented red ginseng (test) or nonfermented red ginseng (reference) extract along with 240 mL of tap water. Any food or water intake was not allowed during the first 4 h after dose. At 4 h after the oral administration, all subjects were given standardized meals. Study individuals were not allowed to remain in a supine position or to sleep until 8 h after the oral administration. For the determination of compound K concentrations, 7 mL of venous blood samples was collected prior to the administration of the dose and at 0.5, 1, 1.5, 2, 3, 4, 5, 6, 8, 10, 12, and 24 h after the administration of the dose at each time period. Each sample was collected in heparinized tubes. The blood samples were centrifuged immediately (3000 rpm, 10 min), and harvested plasma samples were frozen at −80°C until LC-MS/MS analysis.

#### 2.5.1. Tolerability

Adverse effects were monitored throughout the study based on spontaneous reports by volunteers, questioning by investigators, and clinical examinations. The investigators assessed all clinical adverse effects in terms of intensity (mild, moderate, or severe), duration, outcome, and relationship to the study drug.

### 2.6. Pharmacokinetic Study in Rats

Twenty male Sprague Dawley rat (KOATECH, Seoul, Korea) aged 7 weeks were used in this experiment. They were acclimatized for 1 week before randomization into two experimental groups. The animals were housed in a room with a 12-hour/12-hour light/dark cycle, a temperature of 24°C, and a humidity of 50%. During the acclimatization period, animals were fed standard rodent chow (2918C, Harlan Co. Ltd., USA) and water ad libitum. The experimental design was approved by Institutional Animal Care and Use Committee in Chemon Inc. (Serial Number 13-R391). Before administration, the animals were fasted overnight with free access to water. Rats were randomly assigned to receive either (1) 500 mg/kg of nonfermented red ginseng extract (*n* = 10) or (2) 500 mg/kg of fermented red ginseng extract (HYFRG) (*n* = 10). The extracts were suspended in sterilized water immediately before administration and administered orally as a single dose. Plasma samples were obtained before the administration and 0.5, 1, 2, 3, 4, 6, 8, 12, and 24 h after the administration in each period. Blood samples were collected from jugular vein using a syringe into heparinized tubes and centrifuged at 3,000 rpm for 10 minutes. The plasma samples were stored in a −80°C freezer.

### 2.7. Pharmacokinetic Analysis

Individual pharmacokinetic parameters were estimated by Phoenix® WinNonlin® 6.2 (Pharsight Co, CA, USA) using a noncompartmental method. Estimated pharmacokinetic parameters included the area under the plasma drug concentration versus time curve from 0 h to the last measurable concentration (AUC_0–*t*_), maximum plasma drug concentration (*C*_max_), and the time required to reach maximum plasma drug concentration (*T*_max_).

## 3. Results and Discussion 

### 3.1. Enzymatic Transformation of Ginsenosides in Red Ginseng Extract

Ginseng is one of the most commonly used traditional herbal medicines for the treatment of various diseases. In addition, red ginseng contains many bioactive compounds with antioxidant, immunostimulatory, and antiaging activities. Recently published studies suggested that the sugar chains of saponins have been found to be closely related to the biological activity of ginsenosides, so modification of the sugar chains may markedly change the biological activity of ginsenosides [25, 26]. It has been reported that orally administered RG extract did not significantly protect against ischemia reperfusion brain injury, whereas fermented RG did [25]. These results suggested that larger amounts of compound K in fermented red ginseng compared to RG may contribute to the better improvement of ischemic brain injury. Trinh et al. [26] reported more potent inhibitory effects of fermented red ginseng and its primary components compared to RG against hyperlipidemia and hyperglycemia in mice. Based on these results, we evaluated the generation of compound K using commercial enzymes by an HPLC method. As shown in Table 1, the commercial enzymes including Cytolase PCL5, Sumizyme AC, and Rapidase C80Max potently transformed the ginsenosides to compound K after incubation with RG. Among the three enzymes tested, Sumizyme AC produced the largest amount of compound K. Furthermore, the amount of compound K after treatment with a mixture of Cytolase PCL5, Sumizyme AC, and Rapidase C80Max was 0.270 mg/g of RG. Finally, the amounts of compound K, Rg1, and Rb1 were 3.65, 4.12, and 0 mg/g in HYFRG and 0, 1.33, and 6.88 mg/g in RG, respectively.

### 3.2. Method Development and Validation for Compound K

**Figure 2:**
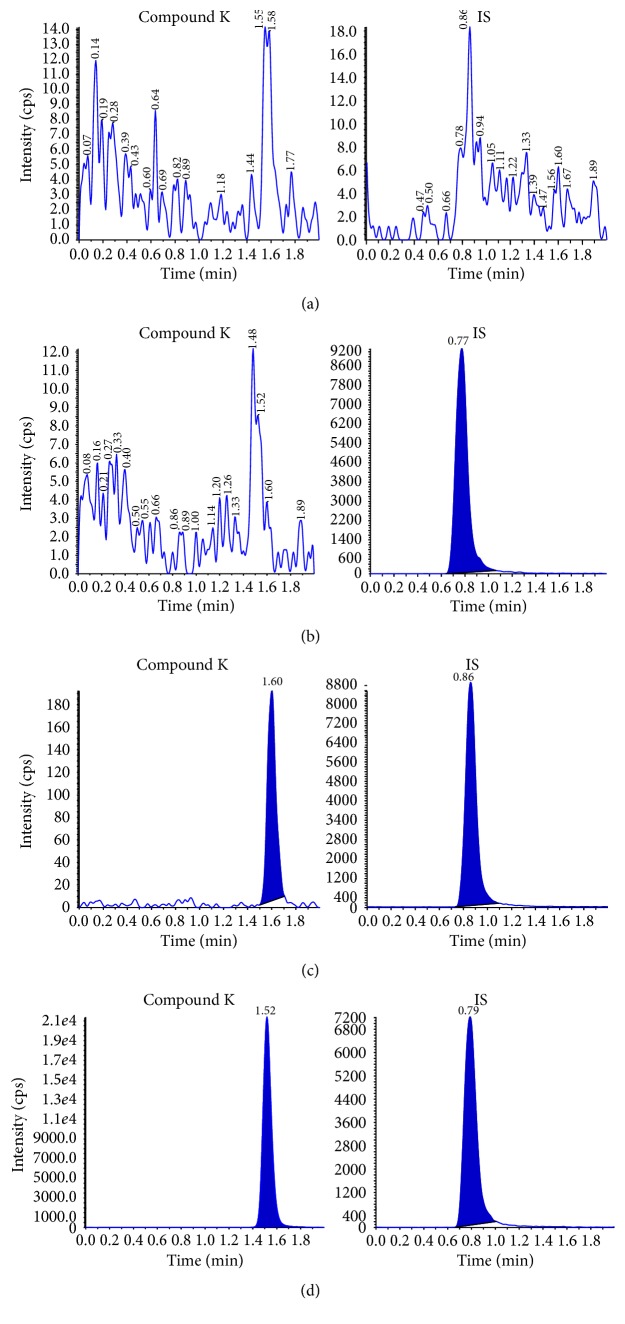
Representative multiple reaction monitoring (MRM) chromatograms of compound K and IS in human plasma. (a) A double blank sample (without compound K and IS); (b) blank sample (without compound K and with IS at 454.55 ng/mL); (c) LLOQ sample (compound K at 1 ng/mL and IS at 454.55 ng/mL); (d) a volunteer sample taken 6 h after administration of 3 g HYFRG, corresponding to a concentration of 115 ng/mL. The retention times of compound K and IS were 1.52 min and 0.79 min, respectively.

LC-MS/MS has high detection specificity because only ions derived from analytes of interest are measured. Using six different lots of human plasma, double blank sample, blank sample, plasma sample spiked with 1 ng/mL of compound K and IS, and volunteer sample collected at 6 h after the administration of 3 g of RG or HYFRG were analyzed. When comparing the chromatograms of samples, the endogenous substances did not interfere with retention times of compound K or IS (Figure 2). The method was validated using the criteria mentioned above and found to have good linearity in the concentration range of 1–1000 ng/mL on five independent runs. The calibration equation with mean (±SD) slope and intercept (*n* = 5) was *y* = 0.01425(±0.002214)*x* − 0.00015(±0.00126) (mean ± SD *r* = 0.9973 ± 0.001191), where *y* is the peak area ratio of the compound K to IS and *x* is the concentration of the compound K. The coefficient of variation was within ±15% at all nominal concentrations. The LLOQ for compound K was 1 ng/mL, and the signal to noise (S/N) ratio was greater than 10. The intra- and interday assay accuracy and precision data are summarized in Table 2. The intraday accuracy ranged from 85.29% to 109.69% with the precision ranging from 2.7 to 12.44%. The interday accuracy ranged from 98.79% to 107.41% with the precision ranging from 6.34 to 12.41%. Values were within accepted limits specified by the KFDA and US FDA for bioanalytical applications (KFDA 2013, USFDA 2013).

Extraction recovery and matrix effect are summarized in Table 3. The mean (±SD) extraction recovery of compound K was 67.16 ± 7.09%, 69.59 ± 3.67%, and 69.01 ± 10.61% for the low (3 ng/mL), middle (300 ng/mL), and high (800 ng/mL) concentration of QC samples, respectively. The mean matrix effects for compound K at low, middle, and high concentration of QC samples were well within acceptable limits (<±20%).

These results suggested that our developed analytical method was reliable and was subject to minimal matrix effects. The predicted concentrations of compound K at 3, 300, and 800 ng/mL samples differed by not more than 15% of the nominal concentrations in a set of stability tests, namely, in injector (20 h), bench-top (17 h), three repeated freeze-thaw cycles, and freezer stability at −80°C for 17 h. The stability results of compound K in human plasma and in methanol demonstrated that deviations in the predicted concentrations of compound K were within the assay variability limits under any of the examined conditions (Table 4). Although several analytical methods have been used to determine compound K concentrations in human plasma with LC-MS/MS using solid phase extraction or protein precipitation [27], we developed the fully validated analytical method optimized for our laboratory settings to meet both KFDA and USFDA guidelines for validating a bioanalytical method [23, 24]. Our analytical method was successfully used to evaluate the comparative pharmacokinetics of two red ginseng HYFRG or RG extracts in Koreans.

### 3.3. Cross Validation

The variability was within ±15% at QC levels between the human and rat samples (data is not shown). This indicated human blood could be used to prepare calibration standards and quality control samples.

### 3.4. Pharmacokinetic Study in Healthy Volunteers

**Figure 3:**
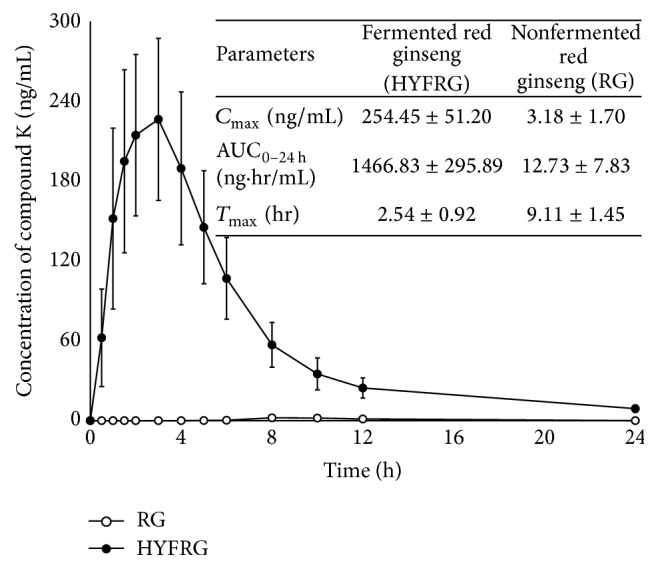
Mean (±SD) plasma concentration-time profile of compound K from 12 healthy Korean male volunteers after a single oral administration of 3 g fermented red ginseng extract (●, HYFRG) and nonfermented red ginseng extract (○, RG). Pharmacokinetic parameters obtained were summarized in insert table.

Our detection method was applied to the pharmacokinetic study of compound K after a single dose of 3 g HYFRG or RG given to 24 healthy Korean male volunteers under fasting state.  Figure 3 shows the profile of mean plasma concentration of compound K versus time in individuals having taken RG and HYFRG. The mean (±SD) AUC_0–*t*_ was 1466.83 ± 295.89 ng·h/mL in the HYFRG treatment group and 12.73 ± 7.83 ng·h/mL in the RG treatment group, respectively. The AUC_0–*t*_ of compound K in the HYFRG treatment group was 115-fold larger than that of the RG treatment group. *C*_max_ of compound K in the HYFRG treatment group was 80-fold higher than that for the RG treatment group (254.45 ± 51.20 ng/mL versus 3.18 ± 1.70 ng/mL). Compound K could be detected in plasma between 1.5 and 4 h after oral administration of HYFRG but 8 and 12 h for RG. The mean (±SD) *T*_max_ of compound K after oral administration of HYFRG and RG was 2.54 ± 0.92 h and 9.11 ± 1.45 h, respectively.

*In a previous study* Shibata and Kim [28, 29] reported before that compound K was detected at 8 h after the oral administration of ginseng powder in healthy men demonstrating variability among individuals. Also recently, we reported differences in the AUC_0–*t*_, *C*_max_, and *T*_max_ for compound K following the oral administration of a fixed dose of fermented and nonfermented ginseng to healthy male subjects [22]. In the fermented ginseng group, AUC_0–*t*_ of compound K was 15.5-fold larger than that of compound K in the nonfermented group (2083.09 ± 91.97 versus 134.50 ± 63.10 ng·h/mL), and the mean *C*_max_ was 27-fold higher compared to the nonfermented group (325.00 ± 91.97 versus 13.88 ± 7.24 ng/mL). *T*_max_ was 3.29 ± 1.00 and 12.04 ± 4.96 h in the fermented ginseng group and nonfermented group, respectively. This fermented ginseng was also prepared in order to increase the amount of compound K (6.3 mg compound K/g, while nonfermented ginseng did not contain compound K). In addition, the pharmacokinetics of compound K after oral administration of 5 g fermented red ginseng extract in healthy men have been reported in another recent study. In this study, *C*_max_, *T*_max_, and AUC_0–*t*_ of compound K were 67.6 ± 39.7 ng/mL, 1.85 ± 0.47 h, and 399.0 ± 260.8 ng·h/mL, respectively [30]. Although our study administered smaller amount of fermented red ginseng compared to the previous study (3 g versus 5 g), our study resulted in higher *C*_max_, longer *T*_max_, and larger AUC_0–*t*_ than the previous study, probably because of the differences in the preparation process of fermented red ginseng. The previous pharmacokinetic* parameters* of compound K after administration of ginseng were well correlated with the amount of compound K in ginseng extracts, especially for samples with and without fermentation. In this study, after fermentation of RG with three enzymes, the amount of compound K in HYFRG (3.65 mg/kg) was 7.3 times higher than RG (0.5 mg/kg), and as a result, its pharmacokinetic parameters of *C*_max_ and AUC_0–*t*_ were 80 and 115 times higher, respectively, than those of RG, indicating enhanced absorption of compound K with HYFRG compared to RG. The increased absorption of compound K observed with HYFRG might be attributed to the fermentation process. However according to our previous study in white ginseng, the fermentation process exerts a lower impact on the absorption of compound K in white ginseng compared to red ginseng [22]. This suggests that the fermentation process may not be the only factor that affects the absorption of compound K. Other factors that might impact the absorption of compound K include the steaming process. Hence future studies are needed to elaborate the differences in the absorption of fermented red and white ginseng.

Also, the absorption of compound K in plasma of individuals receiving HYFRG fermented red ginseng was markedly faster than those in individuals receiving nonfermented red ginseng. These results suggest that compound K in subjects given nonfermented red ginseng has to be formed first as a degradation product of the administered ginsenosides by bacteria present in the large intestine and that it is produced in very small amounts. Therefore direct administration of fermented red ginseng may result in a better absorption which may be associated with more potent pharmacological effects.

### 3.5. Tolerability

In the present study, no serious adverse events were reported, but two subjects experienced diarrhea.

### 3.6. Pharmacokinetic Study in Rats

**Figure 4:**
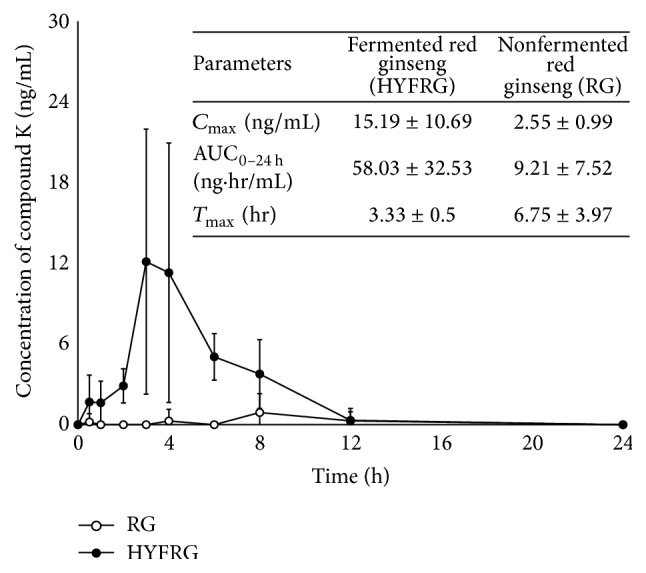
Mean (±SD) plasma concentration-time profile of compound K from 10 male Sprague Dawley rats after a single oral administration of 500 mg/kg fermented red ginseng extract (●, HYFRG) and nonfermented red ginseng extract (○, RG). Pharmacokinetic parameters obtained are summarized in insert table.

We also performed the pharmacokinetic study of compound K in rats. The mean plasma compound K concentration-time profiles after oral administration of 200 mg fermented and nonfermented red ginseng extract to Sprague Dawley rats are shown in Figure 4, and the pharmacokinetic parameters were estimated from these profiles. AUC_0–*t*_ of compound K was 58.03 ± 32.53 ng·h/mL in HYFRG group and 9.21 ± 7.52 ng·h/mL in RG group, respectively. The observed *C*_max_ and *T*_max_ of compound K were 15.19 ± 10.69 ng/mL and 3.33 ± 0.5 h in HYFRG group and 2.55 ± 0.99 ng/mL and 6.75 ± 3.97 h, respectively. Based on these results, we confirmed that the absorption of compound K was also in rats markedly higher and faster for HYFRG compared to RG. Compared to RG, the absorption of compound K with HYFRG was enhanced in rats, but not as much as in humans. This observed difference in the magnitude of absorption enhancement may be attributed to interspecies variability between human and rats. More future studies are needed to address this important issue and to elaborate its influence on the absorption of compound K in order to better estimate the magnitude of the detected variability.

## 4. Conclusions

There is a scarcity of studies that assess the pharmacokinetic profiles of compound K in human and rats after administration of enzyme-treated fermented red ginseng. We describe a rapid and sensitive LC-MS/MS method for analysis of compound K concentrations in human plasma. The developed method fully meets the requirements of the KFDA and USFDA. Additionally, this method has been successfully applied to the pharmacokinetic study of compound K after oral administration of fermented red ginseng (HYFRG) or nonfermented red ginseng (RG) in Korean volunteers and rats. The pharmacokinetic characteristics of compound K, particularly absorption, demonstrated significant differences between HYFRG and RG groups in human and rats. Especially in human the observed tremendous increase in AUC_0–*t*_ and *C*_max_ was out of proportion to the increased amount of compound K administered in HYFRG compared to RG. The prolonged *T*_max_ of compound K observed in the RG group compared to the HYFRG group may be attributed to the preceding intestinal fermentation process of parent ginsenosides to compound K by intestinal microflora before it can be absorbed.

## Figures and Tables

**Table 1:** Amount of compound K obtained during enzymatic transformation of red ginseng extract.

	Sumizyme AC	Rapidase C80max	Cytolase PCL5	Sumizyme AC + Rapidase C80max + Cytolase PCL5
Compound K (mg/g)	0.205 ± 0.003	0.008 ± 0.001	0.122 ± 0.001	0.270 ± 0.002

**Table 2:** Intraday (*n* = 5) and interday (*n* = 3) precision and assay accuracy of quality control samples at four concentrations (1, 3, 300, and 1000 ng/mL) for the determination of compound K concentration in human plasma.

Theoretical concentration (ng/mL)	Acquired concentration (ng/mL) (mean ± SD)		Precision (CV%)		Accuracy (%)	
	Intraday	Interday	Intraday	Interday	Intraday	Interday
1	0.98 ± 0.12	1.02 ± 0.13	12.44	12.41	97.68	102.34
3	2.56 ± 0.17	2.96 ± 0.33	6.66	11.03	85.29	98.79
300	294.15 ± 8.26	322.23 ± 26.97	2.81	8.37	98.05	107.41
1000	1096.86 ± 29.59	1018.66 ± 64.60	2.70	6.34	109.69	101.87

**Table 3:** Extraction recovery and matrix effect of compound K and IS in human plasma (*n* = 3).

	Nominal Concentration (ng/mL)	Extraction recovery		Matrix effect		Process efficiency	
		Mean ± SD (%)	CV (%)	Mean ± SD (%)	CV (%)	Mean ± SD (%)	CV (%)
Compound K	3	67.16 ± 7.09	5.19	86.06 ± 7.45	8.67	57.80 ± 5.12	4.56
	300	69.59 ± 3.67	2.54	95.74 ± 2.21	2.32	66.62 ± 4.23	5.13
	800	69.01 ± 10.61	7.31	87.48 ± 4.64	5.00	63.97 ± 3.89	3.98
(R)-Amlodipine (IS)	454.55	98.36 ± 4.27	4.34	116.68 ± 4.96	4.25	91.80 ± 3.34	3.84

**Table 4:** Results of stability of compound K in human plasma and methanol.

Nominal concentration (ng/mL)	Compound K (*n* = 3)		
	3	300	800
*Working stock solution (%)*			
Room temperature, 6 h	89.08 ± 3.89	89.11 ± 7.13	96.13 ± 7.43
*Compound K in plasma (%)*			
Room temperature, 17 h	110.12 ± 3.01	106.83 ± 3.20	107.54 ± 0.20
4°C, 17 h	112.11 ± 5.08	110.62 ± 2.31	107.40 ± 2.45
−80°C, 17 h	115.04 ± 2.66	108.30 ± 1.60	104.95 ± 1.42
*Freeze-thaw stability (%)*			
Three cycles, −80°C	109.09 ± 7.07	109.70 ± 2.38	106.13 ± 5.46
*Stability of extracted (%)*			
Autosampler (4°C), 20 h	110.43 ± 12.89	102.96 ± 4.83	103.46 ± 3.48

## Abbreviations

FDA: Food and Drug Administration
LLE: Liquid-liquid extraction
MRM: Multiple reaction monitoring
MTBE: Methyl-*tert*-butyl-ether.
